# Bioactivity of novel isoxazole-fused heterocycles: comprehensive antimicrobial, antioxidant activities, SwissADME predictions, molecular docking, and DFT analysis

**DOI:** 10.1007/s11030-025-11180-z

**Published:** 2025-04-17

**Authors:** Mohamed A. M. Abdel Reheim, Moaz M. Abdou, Mohamed S. A. El-Gaby, Mohammad Hasan Al-Omari, Ahmed Abu-Rayyan, Waleed H. Al-Assy, Hala M. Refat, Ahmed A. M. Sarhan, Ibrahim S. Abdel Hafiz

**Affiliations:** 1 Department of Chemistry, Faculty of Science, Arish University, Arish, 45511 Egypt; 2 Egyptian Petroleum Research Institute, Nasr City, 11727 Cairo Egypt; 3 Chemistry Department, Faculty of Science (Boys), Al-Azhar University, Nasr City, 11884 Cairo Egypt; 4 Faculty of Science, Applied Science Private University, Amman, 11931 Jordan; 5 Chemistry Department, Faculty of Science, New Valley University, El-Kharga, 72511 Egypt

**Keywords:** Isoxazole, Antimicrobial activity, Antioxidant activity, Molecular docking, Density Functional Theory (DFT), SwissADME predictions, β-lactamases

## Abstract

Among the foremost goals for organic chemists is to discover novel approaches for the synthesis of a particular heterocyclic and its design. Our approach focused on the vital precursor 4-acetyl-3-phenylisoxazol-5(4*H*)-one **3**, as this molecule has an endocyclic carbonyl function in position 5 adjacent to the substituted acetyl function at site 4. Therefore, compound **3** was a crucial component of many types of fused isoxazole. The investigators provide a straightforward synthesis of fused isoxazole from the following categories: pyrano[3,2-*d*]isoxazole **4 & 6**, isochromeno[4,3-*d*]isoxazole **5**, isoxazolo[4',5':5,6]pyrano[3,4-*c*]pyridine **7**, thieno[3',4':4,5]pyrano [3,2-*d*]isoxazole **8**, pyrazolo[4,3-*d*]isoxazole **10a,b** and** 11a,b**, and isoxazolo[4,5-*c*]pyridazine derivatives **14a,b**. The target compounds and their structures were supported by the results of ^1^H-NMR, IR and mass spectroscopy. Molecular docking studies highlighted strong binding affinities to bacterial enzymes crucial for cell wall synthesis, while DFT calculations provided deep insights into their electronic properties and stability. Additionally, the antioxidant potential of compounds **11a,b** was assessed using DPPH and ABTS assays, showing impressive concentration-dependent activity. Addressing the critical issue of antibiotic resistance, especially due to β-lactamases, molecular docking affirmed the high binding propensity of these derivatives with essential β-lactamase proteins (PDB: 1CK3, 6MU9, and 6W2Z). These findings underscore the promise of isoxazoline derivatives as powerful antimicrobial and antioxidant agents, paving the way for further development in combating bacterial resistance and oxidative stress.

## Introduction

The investigation of heterocyclic compounds has garnered significant attention due to their impressive pharmacological potential, prompting extensive research in recent years. Among these compounds, nitrogen- and oxygen-containing heterocycles have proven exceptionally valuable, making them prime subjects for further study [1–4]. A notable example is the isoxazole ring, an essential pharmacophore characterized by adjacent nitrogen and oxygen atoms [5]. What makes isoxazole particularly intriguing is its widespread use as a key intermediate in the synthesis of numerous bioactive molecules. It plays a crucial role in both naturally occurring and synthetic biologically active compounds, including cycloserine, acivicin, and muscimol, which were initially discovered in microorganisms, higher plants, and marine sponges [6–8].

**Fig. 1:**
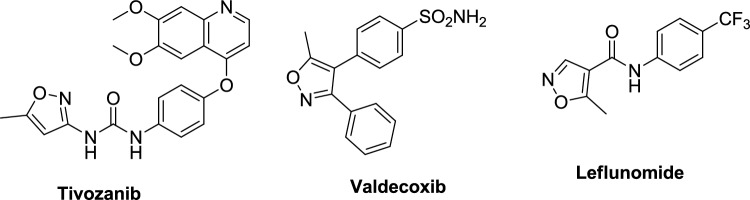
Examples of marketed isoxazole-based pharmaceutical drugs

**Fig. 2:**
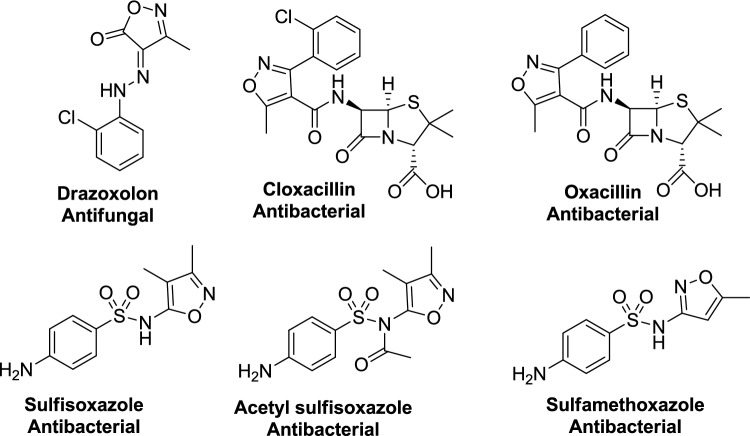
Marketed Antimicrobial drugs featuring the isoxazole nucleus

Isoxazole derivatives exhibit a wide array of therapeutic activities, such as antifungal, antiviral [9], antihistaminic [10], antimicrobial [11], antioxidant [12], anticancer [13], and anti-inflammatory effects. Beyond their medicinal applications, they are utilized in agricultural and industrial [14] settings as insecticides [15], herbicides [16], fungicides [17], and anticorrosive coatings [18]. Several isoxazole-based drugs have successfully entered the market, including valdecoxib, leflunomide, sulfamethoxazole, and the recently approved kinase inhibitor Tivozanib, which received FDA approval in 2021 (Fig. 1). Additionally, a selection of these marketed antimicrobial drugs featuring the isoxazole nucleus is illustrated in Fig. 2.

Furthermore, isoxazolones serve as exceptional precursors for synthesizing other heterocyclic molecules, such as quinolines, imidazoles, oxazinones, and pyridopyrimidines [19–23]. They are also involved in various chemical processes, leading to the formation of unique polycyclic structures and intermediates for biologically active substances, including amino acids, amino alcohols, and amino esters [24–26].

When the isoxazole ring is fused with another ring, it forms a fused isoxazole, which possesses unique structural properties. The five-membered ring introduces specific angles into small molecules, enhancing their interactions with biological targets—a feature less prominent in six-membered rings. This structural flexibility allows medicinal chemists to modify fused isoxazole molecules, thereby improving their pharmacokinetics, potency, and selectivity. As a result, these compounds are highly valued for developing therapeutics with targeted biological functions [27].

Fused isoxazoles demonstrate remarkable biological activities, including analgesic [28], antifungal [29, 30], anti-inflammatory, antioxidant [31], anticancer [32, 33], and antimicrobial effects [34]. Notably, drugs such as paliperidone and risperidone, used to treat schizophrenia and related disorders, as well as zonisamide, an anticonvulsant for partial seizures, are derived from fused isoxazoles [35–37].

A significant challenge in antimicrobial therapy is the activity of β-lactamases, enzymes that confer resistance to β-lactam antibiotics by hydrolyzing the amide bond in the β-lactam ring. This resistance mechanism severely undermines the effectiveness of these widely used antibiotics, complicating the treatment of bacterial infections [38].

Building on our research efforts [39–44], we have synthesized novel fused isoxazoles and evaluated their antioxidant potential, antibacterial efficacy, molecular docking interactions, and Density Functional Theory (DFT) studies. These newly synthesized derivatives were strategically modified to enhance their physicochemical properties and pharmacological activities. Notably, compound 3 plays a pivotal role in various fused isoxazole types. Molecular docking studies provided critical insights into how these compounds interact with target biomolecules, offering valuable information on their antimicrobial and antioxidant mechanisms [45]. Additionally, DFT calculations elucidated the electronic structures and reactivity profiles of these compounds, highlighting their potential as promising therapeutic agents.

## Results and discussion

### Chemistry

**Scheme 1:**
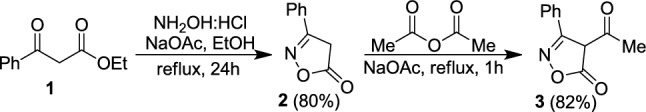
Synthesis of 4-acetyl-3-phenylisoxazol-5(4*H*)-one **3**

The initial compound, 4-acetyl-3-phenylisoxazol-5(4*H*)-one **3**, was synthesized using a two-step process as depicted in Scheme 1. The first step involved a cyclocondensation reaction of ethyl benzoylacetate **1** with hydroxylamine hydrochloride to produce 3-phenylisoxazol-5(4*H*)-one **2** [46]. In the second step, this compound was acetylated using acetic anhydride and sodium acetate under reflux conditions, forming the compound **3** [47].

**Scheme 2:**
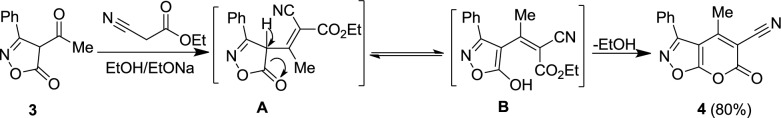
Synthesis of pyrano[3,2-*d*]isoxazole-5-carbonitrile** 4** via Knoevenagel condensation and intramolecular cyclization

The synthesis of 4-methyl-6-oxo-3-phenyl-6*H*-pyrano[3, 2-*d*]isoxazole-5-carbonitrile **4**, an essential precursor for various pyrano[3, 2-*d*]isoxazoles, was efficiently achieved by reacting compound **3** with ethyl cyanoacetate (Scheme 2). This reaction occurred in a 1:1 molar ratio under reflux conditions in ethanol, facilitated by sodium ethoxide. The mechanism is proposed to involve a Knoevenagel condensation forming a transient intermediate that exists as keto (**A**) and enol (**B**) tautomers. Subsequent intramolecular cyclization, accompanied by the loss of ethanol, leads to the final product **4** (Scheme 2).

The structure of **4** was confirmed through various spectroscopic techniques. The IR spectrum of **4** showed characteristic absorption bands at 3059, 2981, 2206, and 1720 cm⁻^1^, corresponding to the CH-aromatic, CH-aliphatic, cyano, and carbonyl (lactone) groups, respectively. The ^1^H-NMR spectrum exhibited a singlet at *δ* 2.65 ppm for the CH₃ group and a multiplet ranging from *δ* 6.96 to 7.97 ppm for the phenyl protons. Furthermore, the mass spectrum (MS) of **4** supported the proposed structure with a molecular ion peak (M peak) at m/z 252, consistent with the molecular formula (MF) C₁₄H₈N₂O₃.

**Scheme 3:**
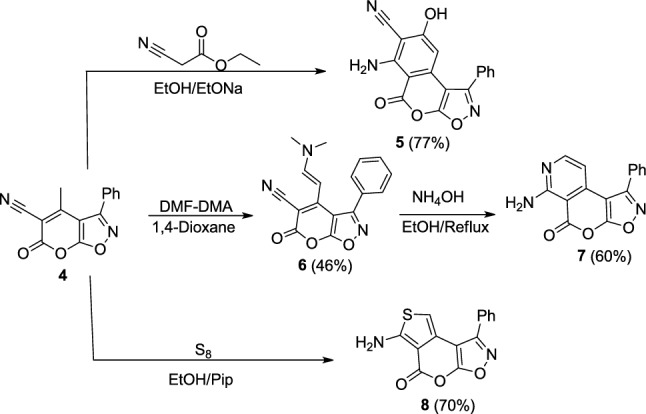
Synthesis of novel pyrano[3,2-*d*]isoxazole derivatives from **4**

Compound **4** is a precursor for various pyrano[3, 2-*d*]isoxazoles **5–8** (Scheme 3). Specifically, the isochromeno[4, 3-*d*]isoxazole derivative, labeled as compound **5**, was synthesized by reacting compound **4** with ethyl cyanoacetate in the presence of sodium ethoxide under reflux conditions. The characteristic newly formed groups, NH_2_ and the phenolic hydroxyl were confirmed by the IR and ^1^H-NMR spectra. The ^1^H-NMR spectrum shows a multiplet at δ 7.50–7.53 ppm attributed to aromatic and NH_2_ protons, and a singlet at *δ* 8.31 ppm corresponding to a hydrogen-bonded hydroxyl group.

Our investigation was expanded to explore the reactivity of **4** towards dimethylformamide dimethylacetal (DMF-DMA) as an electrophile and ethyl cyanoacetate as a carbon nucleophile (Scheme 3). Treatment of compound **4** with DMF-DMA under reflux conditions in dry 1,4-dioxane yielded the pyranoisoxazole **6**. The structural integrity of **6** was confirmed by spectral data, which matched the proposed structure of the pyranoisoxazole derivative **6**. The IR spectrum of **6** revealed absorption bands at 2194 cm^−1^ for the CN group and at 1720 cm^−1^ for the C = O group. The ^1^H-NMR spectrum of compound **6** showed a singlet at *δ* 3.34 ppm due to the methyl of the N(CH_3_)_2_ groups, two doublets at *δ* 6.61 and 7.10 ppm (*J* = 12 Hz and 16 Hz) indicative of olefinic protons, and a multiplet between *δ* 7.20 and 7.95 ppm for the phenyl protons.

The heterocyclization of compound **6**, facilitated by ammonium hydroxide in ethanol, led to a novel isoxazolopyranopyridine **7** (Scheme 3). The structure of **7** was confirmed through spectral data. The IR spectrum of **7** lacked any absorption bands indicative of a nitrile function but displayed characteristic absorption bands at 3424 and 3400 cm^−1^, characteristic of an amino group. The ^1^H-NMR spectrum of **7** revealed a new downfield singlet signal at *δ* 6.60 ppm, corresponding to the –NH_2_ group. Additionally, the MS of **7** showed a M peak at m/z 279 (M^+^), corroborating its molecular weight.

Further extending the study, compound **4** was reacted with elemental sulfur in refluxing ethanol in the presence of piperidine, following Gewald's synthesis, which produced thienopyranoisoxazol-6-one **8** (Scheme 3). The IR spectrum of **8** displayed absorption bands at 3425 and 3400 cm^−1^, characteristic of the amino group, and confirmed the absence of a nitrile function. The ^1^H-NMR spectrum of **8** showed the lack of methyl signal and a singlet signal at 7.30 ppm due to NH_2_ protons.

**Scheme 4:**
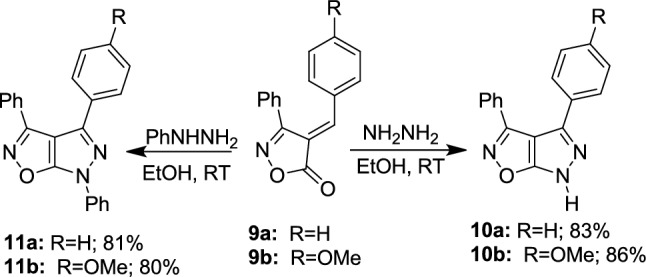
Reactivity of alkylidene isoxazol-5-ones **9a,b** towards hydrazines as nucleophiles

Next, the chalcone moiety of alkylidene isoxazol-5-ones **9a,b** [48] facilitated the exploration of their reactivity towards hydrazines, serving as a binucleophile (Scheme 4). Novel pyrazolo[4,3-*d*]isoxazoles **10a,b** were synthesized by reacting compound **9a,b** with hydrazine hydrate in ethanol at room temperature (RT). The structural confirmation of compounds **10a,b** was achieved through spectral data. For instance, the MS of **10a** exhibited a M peak at m/z 261, consistent with a MF of C_16_H_11_N_3_O. Its IR spectrum did not display any absorption bands indicative of a C=O group. The ^1^H-NMR spectrum of compound **10b** aligned with its predicted structure, showing a signal at *δ* 3.83 ppm for methoxy protons, a multiplet between *δ* 7.04 and 7.82 ppm for phenyl protons, and a downfield signal at *δ* 8.63 ppm, attributed to the NH proton of the pyrazole nucleus.

Similarly, the corresponding pyrazolo[4,3-*d*]isoxazole derivatives **11a,b** were synthesized by reacting **9a,b** with phenyl hydrazine in ethanol at RT (Scheme 4). The IR spectra of **11a,b** showed no peaks for the conjugated carbonyl group, indicating its reaction. Analytical data for **11b** revealed a MF of C_23_H_17_N_3_O_2_, with a M peak at M^+^ 367. The ^1^H-NMR spectrum of **11b** showed a signal at *δ* 3.86 ppm attributable to the methoxy group and a multiplet between *δ* 6.90 and 7.72 ppm for the phenyl protons.

**Scheme 5:**
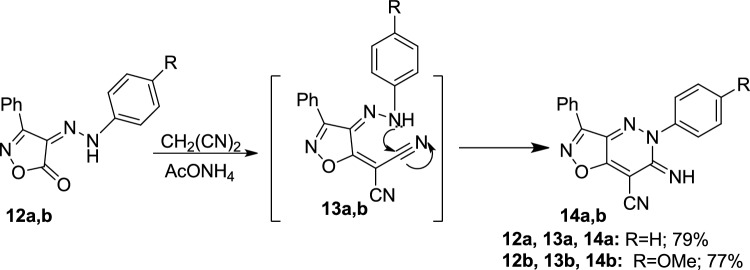
Formation of **14a,b** via Knoevenagel condensation and intramolecular cyclization

The isoxazolo[4,5-*c*]pyridazine-7-carbonitrile derivatives **14a,b** were synthesized by reacting **12a,b** [49] with malononitrile in the presence of ammonium acetate (Scheme 5). The synthesis of **14a,b** is theorized to proceed via a Knoevenagel condensation reaction, forming a non-isolable acyclic intermediate **13a,b**. Subsequent intramolecular cyclization occurs when the NH group of the hydrazone adds to the cyano group, completing the synthesis as illustrated in Scheme 5.

The molecular structures of these novel compounds were confirmed by spectral data. The IR spectra of **14a,b** revealed absorption bands characteristic of the -NH and the nitrile group –CN function, confirming their presence in both structures. The bands appeared at 3311 cm^−1^ and 2204 cm^−1^ for **14a**, and at 3331 and 2194 cm^−1^ for **14b**, respectively. Specifically, the MF of **14b** was identified as C_19_H_13_N_5_O_2_ (M^+^ 343) based on analytical data. Its ^1^H-NMR spectrum showed an NH proton in the downfield region at *δ* 8.46 ppm, which could be exchanged by shaking with D_2_O, alongside multiplet signals for phenyl protons and a singlet at *δ* 3.85 ppm corresponding to methoxy protons.

### DFT computations

**Fig. 3:**
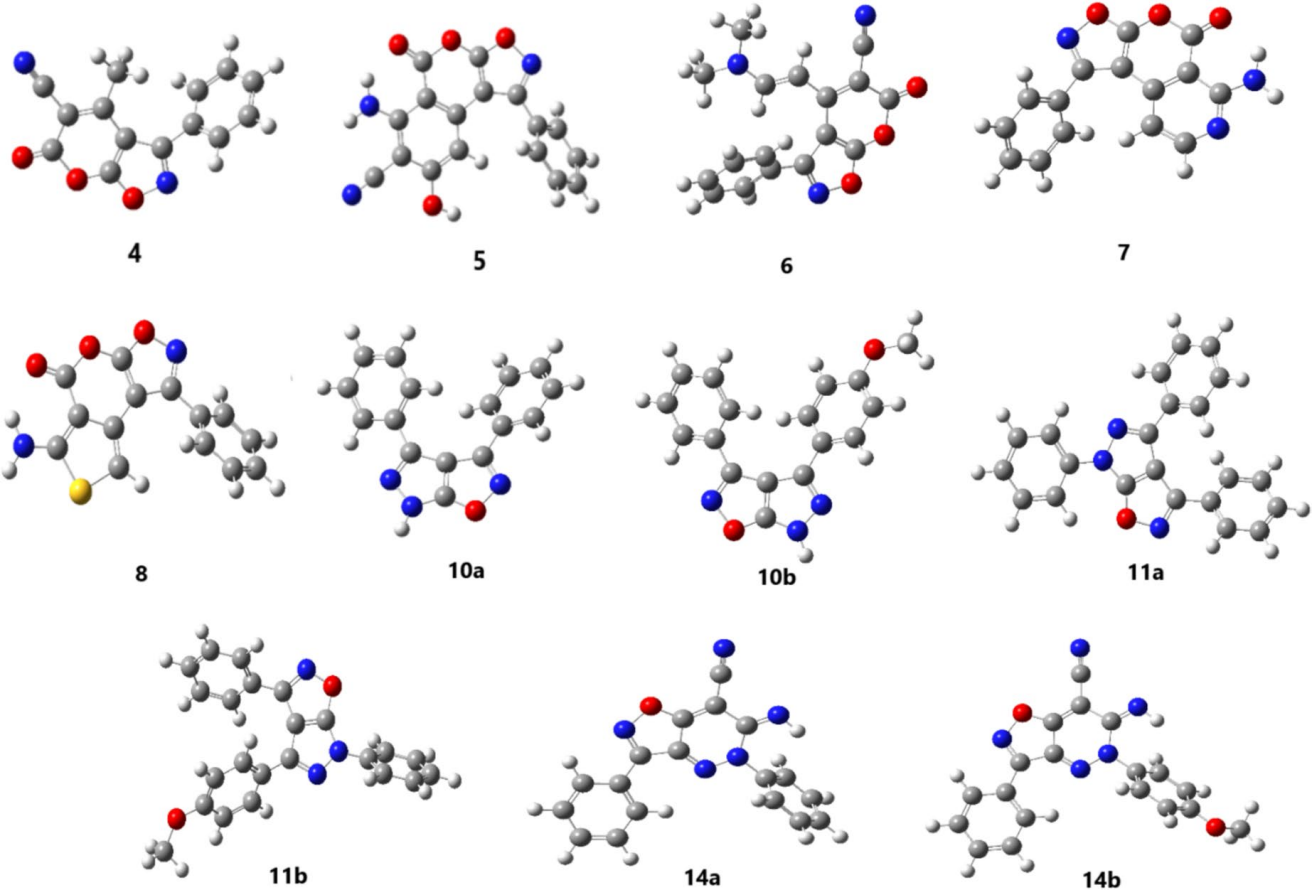
The structure of optimization of the **4**^**_**^**8, 10a,b, 11a,b** and **14a,b** derivatives

To gain some information about the structures of the compounds **4**^**_**^**8, 10a,b, 11a,b** and **14a,b**, a molecular modeling investigation was conducted using the Gaussian 09 W software through B3LYP/6-311G* (*d,p*) (Fig. 3). Quantum chemical calculations, based on the frontier molecular orbitals (HOMO and LUMO), can predict the molecular system, chemical properties, and chemical behavior of compounds. These calculations also reveal how the molecule interacts with other molecules [50–54]. The HOMO–LUMO energy gap is a useful indicator of the molecule's kinetic stability and chemical reactivity [55–57]. Moreover, they are employed in the fields of molecular electronics CT, light excitation, donating capacity, and site selectivity. A molecule with a narrow gap is more polarized and is considered to be a soft molecule, which tends to have higher reactivity but poorer kinetic stability. Conversely, a molecule with a wider gap is considered to be a hard molecule, which exhibits lesser reactivity. The global reactivity characteristics, including electronegativity (*χ*), chemical potential (*μ*), global hardness (*η*), global softness (*S*), and electrophilicity index (*ω*), are determined by evaluating the energies of frontier molecular orbitals.

$$ \begin{gathered} \chi = - {1}/{2}\left( {{\mathrm{E}}_{{{\mathrm{HOMO}}}} + {\text{ E}}_{{{\mathrm{LUMO}}}} } \right), \hfill \\ \mu = \chi = { 1}/{2}\left( {{\mathrm{E}}_{{{\mathrm{HOMO}}}} + {\text{ E}}_{{{\mathrm{LUMO}}}} } \right), \hfill \\ \eta = {1}/{2}\left( {{\mathrm{E}}_{{{\mathrm{HOMO}}}} - {\text{ E}}_{{{\mathrm{LUMO}}}} } \right), \hfill \\ {\mathrm{S}} = {1}/{2}\eta , \hfill \\ \omega = \mu^{{2}} /{2}\eta . \hfill \\ \end{gathered} $$

**Fig. 4:**
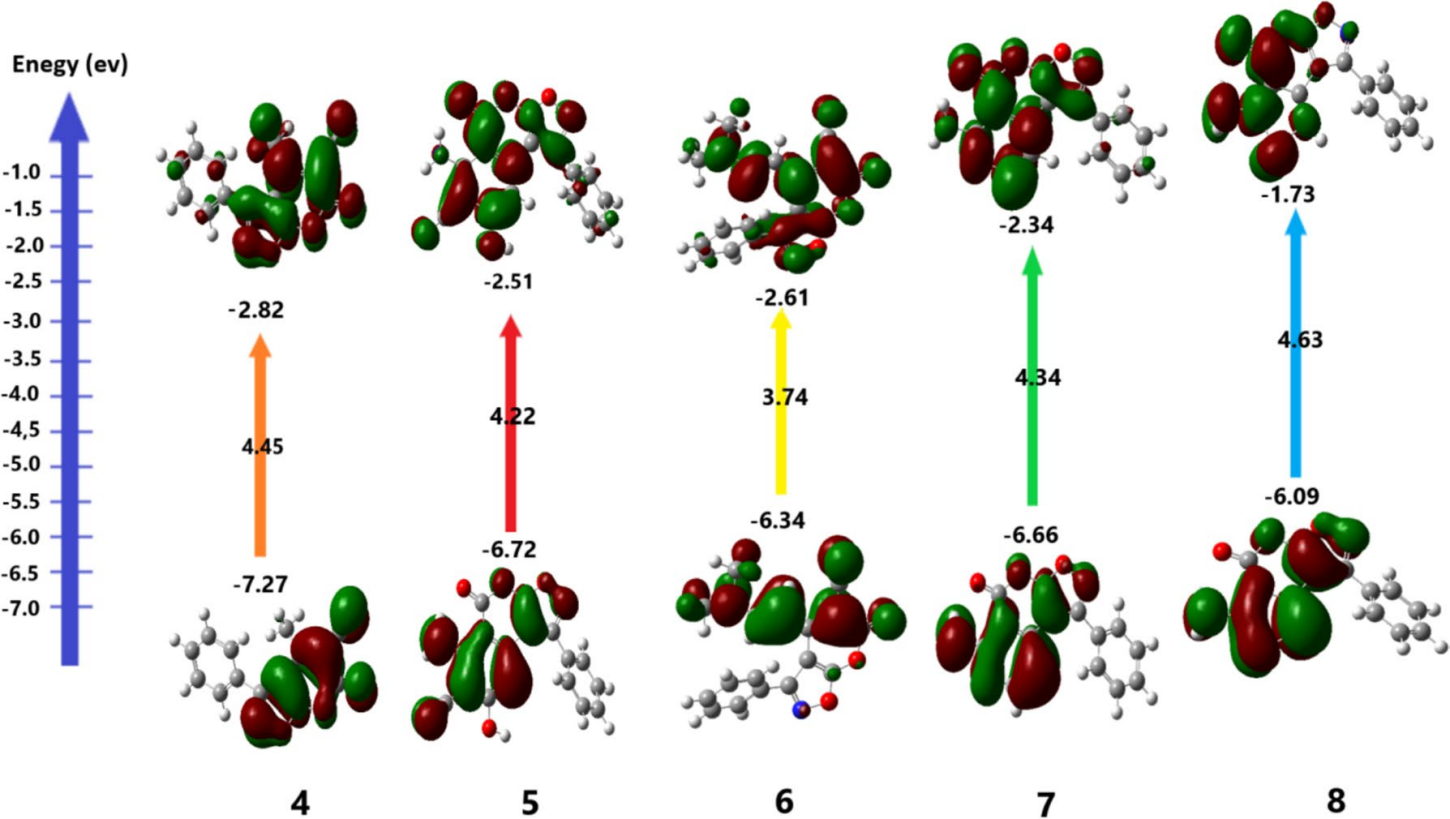
Frontier orbital energies of derivatives **4**^**_**^**8**

**Fig. 5:**
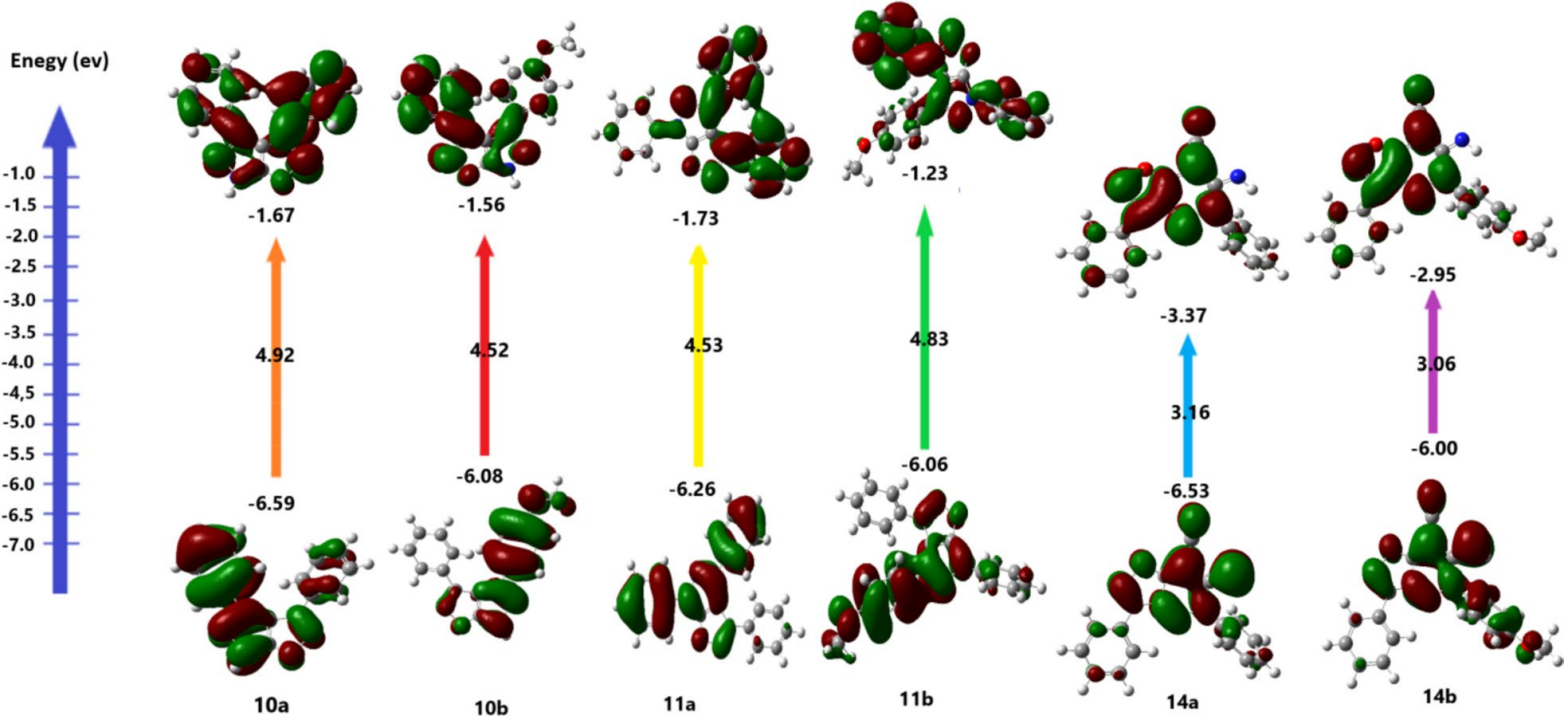
Frontier orbital energies of derivatives **10a,b, 11a,b** and** 14a,b**

To investigate the chemical behavior of the synthesized derivatives **4**^**_**^**8, 10a,b, 11a,b** and **14a,b**, we systematically assessed their global and local reactivity parameters. Specifically, the calculated values for μ, χ, S and ω (Table 1). Additionally, the HOMO–LUMO energy gap of the **4**^**_**^**8, 10a,b, 11a,b** and **14a,b** has been visually depicted in Figs. 4 and 5. The results found that **14a,b** and **6** have the lowest optical energy gap EHOMO-LUMO (3.059, 3.158 and 3.73) than other compounds due to the present CN function group. A narrow bandgap enhances the optical characteristics of the products, enabling them to absorb the majority of incident light. Furthermore, the chemical hardness values of **14a,b** and **6** are lesser (1.529, 1.579 and 1.868, respectively) compared to other synthesized compounds. Thus, the three compounds are found to be more reactive.

**Table 1:** Calculated values for global and local reactivity parameters for **4**^**_**^**8, 10a,b, 11a,b** and **14a,b** (H = *HOMO*; L = *LUMO*)

Compounds	Energy (Kcal/mo)	*E*_*H*_ (eV)	*E*_*L*_ (eV)	*E*_*Gap*_ (eV)	*χ* (eV)	*μ* (eV)	*η* (eV)	*ω* (eV)	*δ* (eV)	Dipole (debye)
**4**	− 5.48 × 10^5^	− 7.273	− 2.823	4.450	5.048	− 5.048	2.225	5.727	0.225	7.36
**5**	− 7.02 × 10^5^	− 6.723	− 2.508	4.215	4.615	− 4.615	2.108	5.054	0.237	5.80
**6**	− 6.56 × 10^5^	− 6.341	− 2.606	3.735	4.473	− 4.473	1.868	5.357	0.268	12.61
**7**	− 6.07 × 10^5^	− 6.662	− 2.322	4.341	4.492	− 4.492	2.170	4.649	0.230	4.60
**8**	− 7.98 × 10^5^	− 6.088	− 1.733	4.355	3.911	− 3.911	2.178	3.512	0.230	6.27
**10a**	− 5.37 × 10^5^	− 6.588	− 1.668	4.920	4.128	− 4.128	2.460	3.464	0.203	1.68
**10b**	− 6.09 × 10^5^	− 6.077	− 1.558	4.520	3.818	− 3.818	2.260	3.224	0.221	2.98
**11a**	− 6.82 × 10^5^	− 6.259	− 1.733	4.526	3.996	− 3.996	2.263	3.529	0.221	1.86
**11b**	− 7.54 × 10^5^	− 6.059	− 1.231	4.828	3.645	− 3.645	2.414	2.752	0.207	3.31
**14a**	− 6.54 × 10^5^	− 6.529	− 3.371	3.158	4.950	− 4.950	1.579	7.760	0.317	9.78
**14b**	− 7.25 × 10^5^	− 6.004	− 2.946	3.059	4.475	− 4.475	1.529	6.547	0.327	9.94

**Fig. 6:**
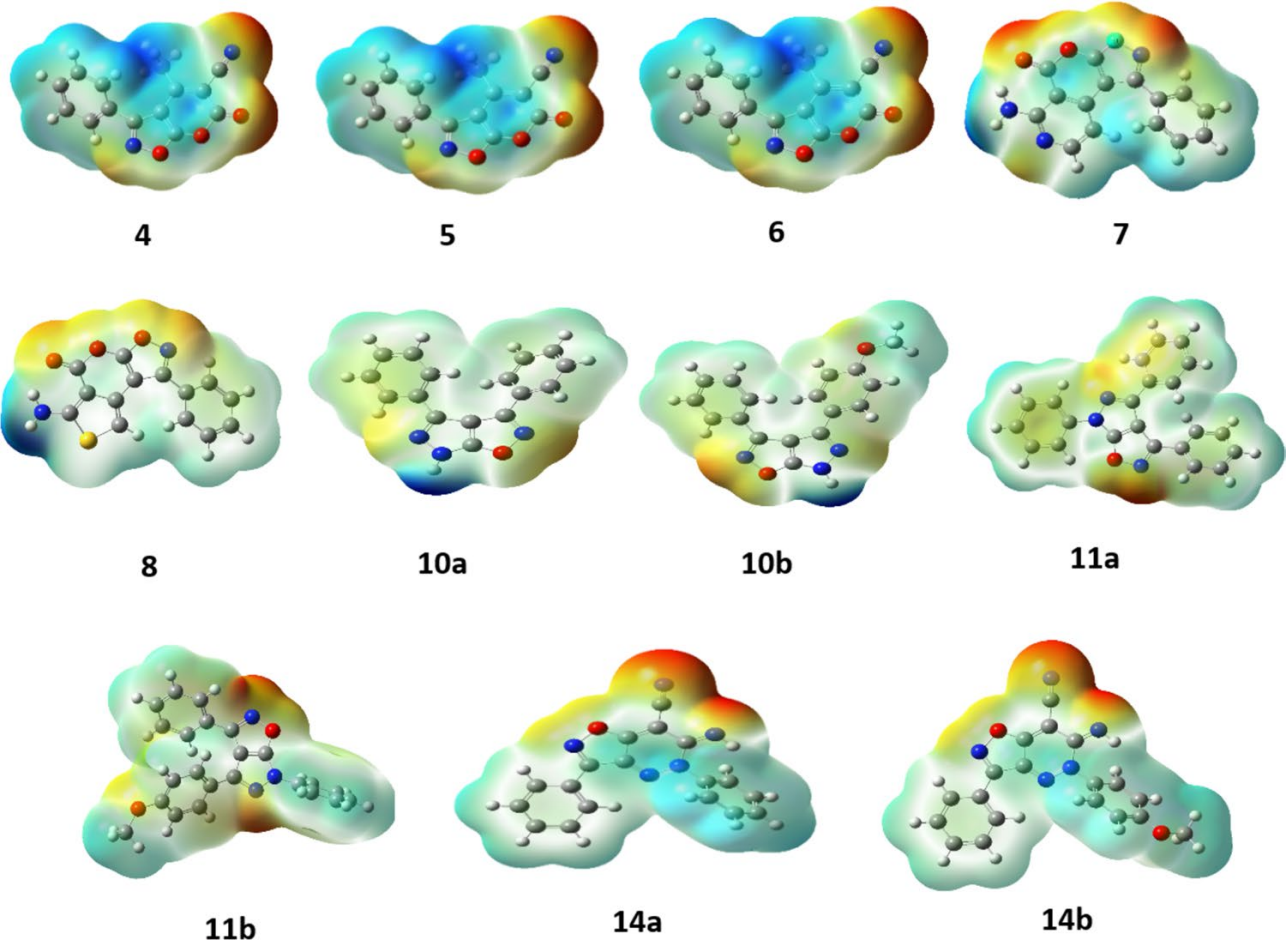
Molecular electrostatic potential of the synthesized **4**^**_**^**8, 10a,b, 11a,b** and **14a,b**

Electrostatic potential energy maps (EMP) or electrostatic surface potential (ESP) maps depict the charge distributions in three dimensions across molecules [58]. Molecular electrostatic potential (MEP) offers a graphical approach for comprehending the polarity of a molecule's reactivity. The negative electrostatic potential arises from the attractive interaction between protons and the electron density within molecules. Conversely, regions characterized by low electron density lead to high electrostatic potential, resulting from proton repulsion by atomic nuclei. The electrostatic potential surface is depicted in Fig. 6; it was calculated utilizing DFT in conjunction with the 6-311G*(d,p) basis set and the B3LYP exchange–correlation functional.

Various colors are used to denote the distinct values of electrostatic potential. For instance, regions with the highest positive potential are colored blue, while areas with the lowest potential are indicated by green. Per Fig. 6, we have noticed a higher negative electrostatic potential in the isoxazolone ring, which is fused with hetero rings. These rings have a greater number of oxygen and nitrogen atoms and functional groups such as CN, which contribute to cyclization and reactivity towards electrophiles. The function groups –CH_3_, –NH_2_, –NH, and –N(CH_3_)_2_ linked to the rings exhibit a higher positive electrostatic potential. The hydrogen atoms bonded to the heterocyclic rings possess a potential energy of zero.

### Antimicrobial activity

The antimicrobial efficacy of synthetic compounds **4**^_^**8**, **10a,b**, **11a,b**, and **14a,b** were evaluated using standard drugs (Ampicillin for bacteria and Clotrimazole for fungi) as controls. The tests were conducted against four microbial species: *E. coli*, *B. megaterium*, *B. subtilis*, and *T. harzianum* (Table 2). The efficacy is assessed by the zone of inhibition in millimeters, percentage activity index, and minimum inhibitory concentration (MIC) in µg/mL.

**Table 2:** Antimicrobial efficacy of synthesized compounds **4**^**_**^**8, 10a,b, 11a,b** and **14a,b**

Compounds	*E.coli*			*B. megaterium*			*B. subtilis*			*T. harzianum*		
	Inhibition (mm)	% Activity	MIC (µg/mL)	Inhibition (mm)	% Activity	MIC (µg/mL)	Inhibition (mm)	% Activity	MIC (µg/mL)	Inhibition (mm)	% Activity	MIC (µg/mL)
**4**	12	52.17	15	15	65.22	15	20	86.96	75	12	54.55	75
**5**	15	65.17	35	20	86.96	75	15	65.22	75	15	68.18	75
**6**	15	65.17	75	20	86.96	15	15	65.17	75	15	45.45	125
**7**	12	52.17	75	15	65.22	35	12	52.17	75	10	45.45	125
**8**	12	52.17	75	20	86.96	15	12	52.17	75	10	45.45	75
**10a**	20	86.96	35	15	65.22	75	20	86.96	75	20	90.91	125
**10b**	15	65.22	35	15	65.22	75	15	65.22	75	12	54.55	125
**11a**	20	86.96	15	21	90.30	35	20	86.96	35	18	81.81	75
**11b**	20	86.96	15	21	90.30	35	20	86.96	35	20	90.91	75
**14a**	15	65.22	35	15	65.22	35	15	65.22	35	15	68.18	35
**14b**	12	52.17	75	12	52.17	35	15	65.22	75	15	68.18	35
Ampicillin	23	100.00	12	23	100.00	12	23	100.00	12	–	–	–
Clotrimazole	–	–	–	–	–	–	–	–	–	22	100.00	12

In the case of *E. coli*, compounds **11a**,**b** demonstrate a high percentage of activity (86.96%) with a significant inhibition zone of 20 mm, similar to Ampicillin, which shows an activity index of 100% and an inhibition zone of 23 mm. The MIC values for *E. coli* are lowest for Ampicillin (12 µg/mL), indicating its superior efficacy. For *B. megaterium*, compounds **11a,b** were most effective, each displaying a high activity index of 90.30% and an inhibition zone of 21 mm. Their MIC values (35 µg/mL) are among the lowest observed for *B. megaterium*, with only three of the eleven tested compounds exhibiting higher MIC values. This suggests that compounds **11a,b** demonstrate notable antimicrobial potency against this bacterial strain.

Similarly, against *B. subtilis*, compounds **11a**,**b** show a consistent pattern, achieving a high activity index and inhibition zone comparable to those against *B. megaterium*, with an equally low MIC of 35 µg/mL. This suggests their robust antimicrobial properties. In contrast, for *T. harzianum*, compound **11b** stands out with the highest activity index (90.91%) and an inhibition zone of 20 mm. However, the MIC values for this fungus are generally higher, with several reaching 125 µg/mL, indicating a reduced sensitivity compared to the bacterial species.

These observations suggest that the response of the tested microbial species to the synthetic compounds varies, with some like **11a**,**b** showing high activity across multiple species, potentially indicative of broad-spectrum antimicrobial properties. Compounds **11a,b** demonstrate antimicrobial activity comparable to that of the standard drugs against the tested targets, highlighting their potential as broad-spectrum antimicrobial candidates**.**

### Antioxidant activity

**Fig. 7:**
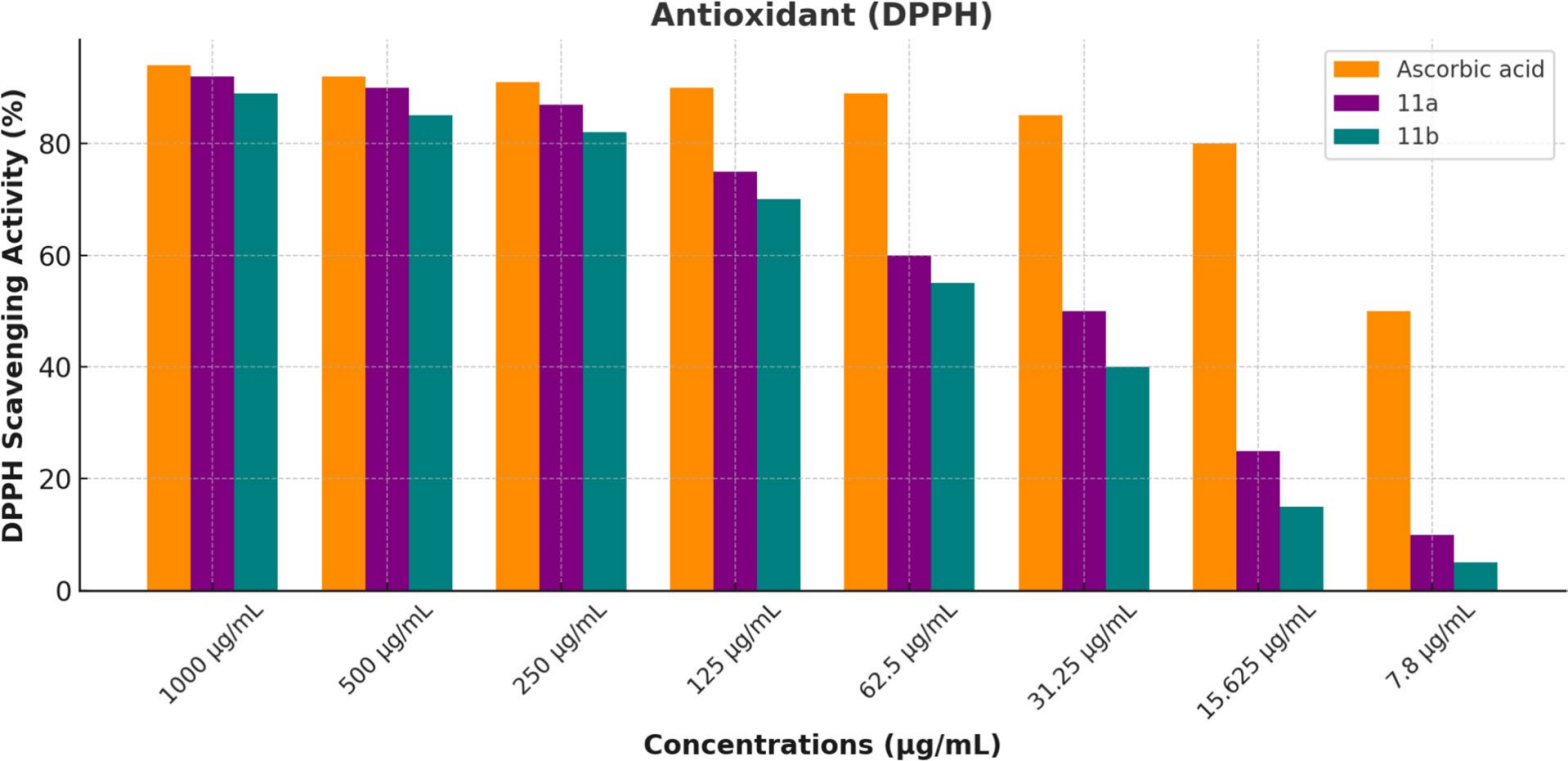
Antioxidant activity of **11a**,**b** at various concentrations by the DPPH method

The antioxidant potency of **11,b** was evaluated using the DPPH (2,2-diphenyl-1-picrylhydrazyl) radical scavenging activity assay at various concentrations ranging from 1000 µg/mL to 7.8 µg/mL [59]. The results in Fig. 7 indicate that both compounds exhibited concentration-dependent antioxidant activity against the DPPH free radicals. At the highest concentration tested (1000 µg/mL), both compounds demonstrated high antioxidant activity, with compound **11a** showing slightly higher effectiveness than **11b**. As the concentration decreased, the scavenging activity of both compounds also declined. However, compound **11a** consistently maintained higher activity than **11b** across all concentrations. Ascorbic acid, used as a positive control, showed the highest scavenging activity overall, with an IC_50_ value of 7.61 µg/mL, compared to 35.5 µg/mL for compound **11a** and 45.6 µg/mL for compound **11b**. These findings highlight that while compounds **11a**,**b** possess significant antioxidant properties, ascorbic acid remains the most potent antioxidant. It is worth noting that other compounds, **4**^**_**^**8**, **10a,b**, and **14a,b**, did not demonstrate any significant efficiency in the DPPH assay, thus their data were excluded from the analysis.

### SwissADME predictions

This study employed Swiss ADME to analyze the physicochemical, pharmacokinetic, and drug-like of synthetic compounds **4**^**_**^**8, 10a,b, 11a,b** and **14a,b** (Tables 3, 4). The compounds varied significantly in lipophilicity, with higher values suggesting better membrane permeability and a potential increase in non-specific binding.

**Table 3:** The physicochemical, pharmacokinetic, drug-like and related parameters of **4, 5, 6, 7, 8, 10a** and **10b**

Compounds		4	5		6	7	8		10a		10b
*Lipophilicity*											
iLOGP		1.94	1.72		2.24	1.85	2.25		2		2.18
XLOGP3		2.20	3.26		2.38	2.61	3.36		3.66		3.63
*Water solubility*											
ESOL Log S	− 3.31		− 4.39	− 3.53		− 3.82		− 4.32		− 4.37	− 4.41
ESO solubility (mg/mL)	1.24e−1		1.29e−02	9.08e−02		4.23e−02		1.36e−02		1.11e−02	1.14e−02
ESOL class	Soluble		Moderately soluble	Soluble		Soluble		Moderately soluble		Moderately soluble	Moderately soluble
*Pharmacokinetics*											
GI absorption		High	High		High	High	High		High		High
BBB permeant		No	No		No	No	No		Yes		Yes
P-gp substrate		No	No		No	No	No		Yes		Yes
CYP1A2 inhibitor		Yes	Yes		Yes	Yes	Yes		Yes		Yes
CYP2C19 inhibitor		No	No		Yes	No	Yes		Yes		Yes
CYP2C9 inhibitor		No	No		Yes	No	No		No		No
CYP2D6 inhibitor		No	No		No	No	No		No		Yes
CYP3A4 inhibitor		No	No		No	No	No		No		No
Skin permeation log Kp (cm/s)		− 6.28	− 5.93		− 6.48	− 6.15	− 5.65		− 5.30		− 5.50
*Drug-likeness*											
Lipinski #violations		0	0		0	0	0		0		0
Ghose #violations		1	1		1	1	1		1		1
Veber #violations		1	1		1	1	1		1		1
Egan #violations		1	1		1	1	1		1		1
Muegge #violations		1	1		1	1	1		1		1
Bioavailability score		0.55	0.55		0.55	0.55	0.55		0.55		0.55

**Table 4:** The physicochemical, pharmacokinetic, drug-like and related parameters of** 11a,b, 14a** and **14b**

Compounds	11a	11b	14a	14b
*Lipophilicity*				
iLOGP	3.46	3.66	2.55	2.86
XLOGP3	5.33	5.3	2.8	2.77
*Water solubility*				
ESOL Log S	− 5.83	− 5.88	− 4.06	− 4.11
ESOSolubility (mg/mL)	4.97e−− 04	4.84e−− 04	2.72e−02	2.64e−02
ESOL Class	Moderately soluble	Moderately soluble	Moderately soluble	Moderately soluble
*Pharmacokinetics*				
GI absorption	High	High	High	High
BBB permeant	Yes	Yes	No	No
P-gp substrate	Yes	Yes	No	No
CYP1A2 inhibitor	Yes	No	Yes	Yes
CYP2C19 inhibitor	Yes	Yes	No	No
CYP2C9 inhibitor	No	Yes	No	Yes
CYP2D6 inhibitor	No	No	No	No
CYP3A4 inhibitor	No	No	No	Yes
Skin permeation log Kp (cm/s)	− 4.57	− 4.78	− 6.22	− 6.43
*Drug-likeness*				
Lipinski #violations	1; MLOGP > 4.15	0	0	0
Ghose #violations	1	1	1	1
Veber #violations	1	1	1	1
Egan #violations	1	1	1	1
Muegge #violations	0; XLOGP3 > 5	0; XLOGP3 > 5	1	1
Bioavailability score	0.55	0.55	0.55	0.55

Water solubility ranged from soluble to moderately soluble, with compounds **11a**,**b** showing notably low solubility, which might impact their pharmacokinetic profiles but could be addressed in drug formulation. All compounds demonstrated high gastrointestinal absorption, which is beneficial for oral drugs.

Regarding drug-likeness, most compounds met Lipinski's rule of five, indicating favorable drug-like properties. However, some violations in Ghose and Veber rules were noted, potentially affecting bioavailability. Despite this, the bioavailability scores were moderately good at 0.55, suggesting these compounds are promising candidates for further optimization and experimental validation to enhance their therapeutic efficacy.

### In silico* molecular docking simulation*

In fact, β-lactamases are crucial in bacterial resistance to β-lactam antibiotics [38]. These enzymes are the leading cause of β-lactam antibiotic inactivation by breaking down the β-lactam ring in antibiotics. β-lactamases disrupt the antibiotic ring, rendering it inactive and preventing it from binding to its target, penicillin-binding proteins (PBPs). PBPs, which are membrane-anchored, facilitate the cross-linking of cell walls via their trans-peptidase activity. Resistance arises when the antibiotic loses its ability to inhibit cell wall synthesis. Several Gram-negative bacteria have naturally occurring β-lactamases that counteract various β-lactam antibiotics. While Gram-negative bacteria are more likely to produce β-lactamases, recent findings indicate that Gram-positive bacteria can also produce these enzymes.

A big problem with β-lactamases in clinical practice is that they can make routinely used antibiotics useless [60]. Methods for overcoming this resistance encompass utilizing β-lactamase inhibitors alongside β-lactam antibiotics and their impact on bacteria. Through molecular docking and theoretical studies, we investigate three types of β-lactamase families in three different bacteria—*E.coli, B.megaterium,* and *B.subtilis*—to develop antibiotics resistant to these families. To comprehend the mechanism, molecular docking was used to examine the technical contact between the vehicles (**10a**, **11a**, and **11b**) and β-lactamases obtained from the Protein Data Bank (PDB: 1CK3, 6MU9 and 6W2Z) [60].

**Fig. 8:**
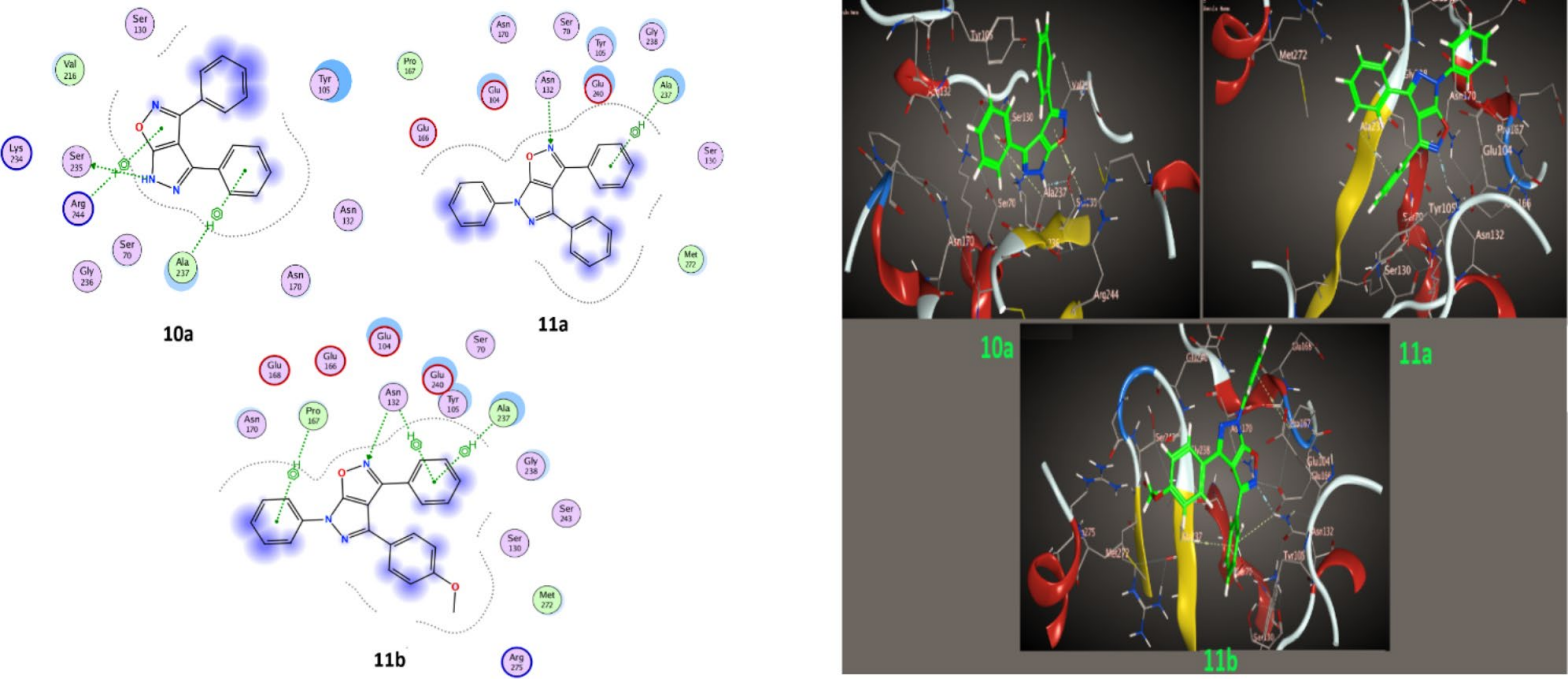
2D, 3D Binding models of **10a**,** 11a,** and** 11b** with a *E. coli* (PDB: 1CK3)

**Fig. 9:**
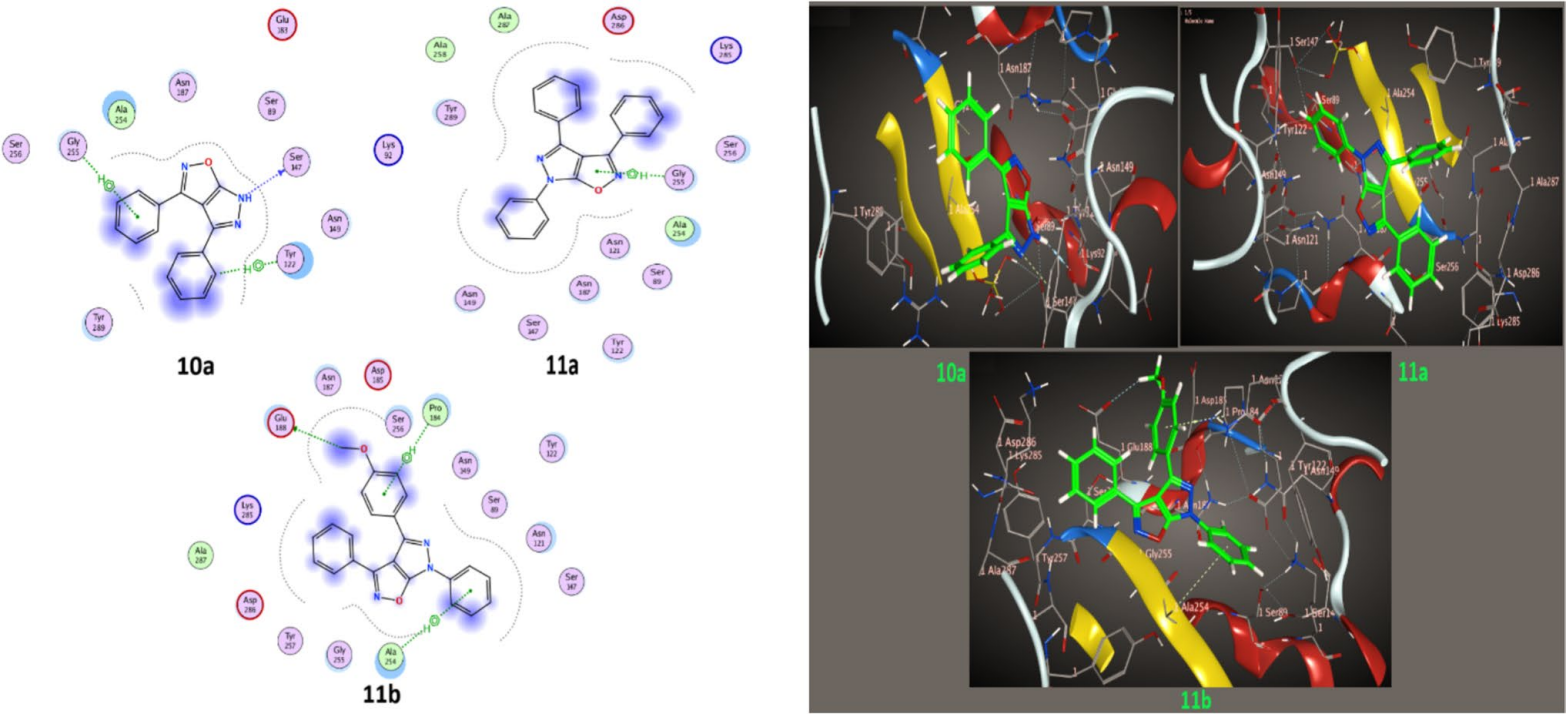
2D, 3D Binding models of **10a**,** 11a,** and** 11b** with a *B. megaterium* (PDB: 6MU9)

**Fig. 10:**
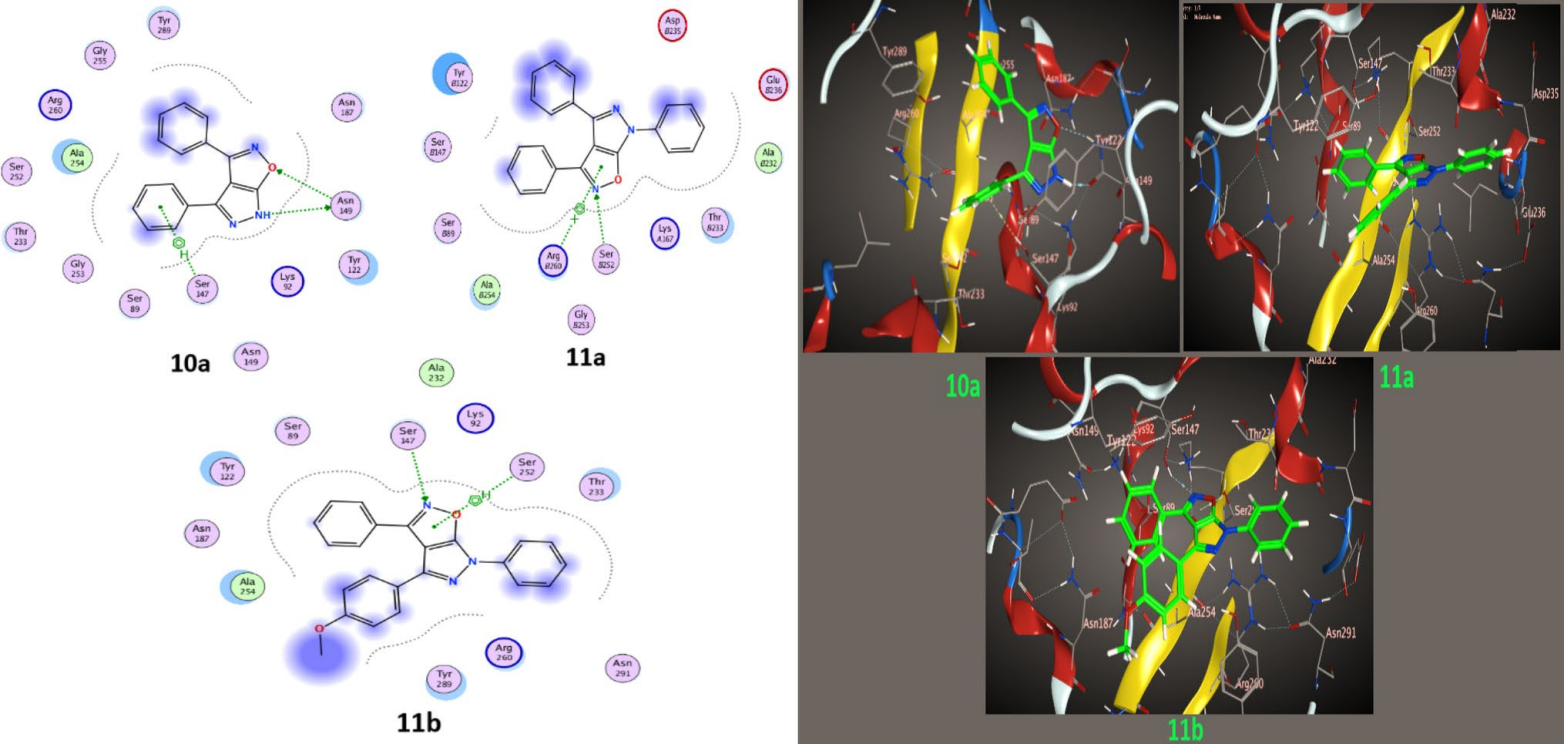
2D, 3D Binding models of **10a**,** 11a,** and** 11b** with a *B. subtilis* (PDB: 6W2Z)

Per the experimental data, the molecular docking study included compound **10a, 11a,b** ligands that yielded promising results when tested against different protein receptors: *E. coli*, *B. megaterium* and* B. subtilis,* as depicted in Figs. 8, 9 and 10 and Table 5. For the* E. coli* (1CK3), the compounds showed docking scores ranging from − 5.18 to − 5.88 kcal/mol, suggesting a substantial affinity towards the targeted enzymes. For *B. megaterium* (6MU9), the docking scores ranged from − 5.18 to − 5.88 kcal/mol, suggesting a substantial binding affinity towards the targeted enzymes. Similarly, for *B. megaterium* (6MU9) under another assessment, the scores ranged from − 5.11 to − 6.37 kcal/mol, indicating a strong binding potential. Against *B. subtilis* (6W2Z), the docking scores ranged from − 5.21 to − 5.88 kcal/mol, reflecting a high interaction potential with the target proteins. The present moiety of isoxazole-pyrazole five hetero-ring, in addition to twisted phenyl rings, gave good binding affinity with target proteins through pi-donor hydrogen bonds, H-Donor bonds and electrostatic pi-cation, and this agrees with experimental data.

**Table 5:** Interaction parameters of the studied compounds with β-lactamases(PDB:1CK3, 6MU9 and 6W2Z) Protein

Selected biomolecules	Ligand	Receptor (Interacting residues)	Interaction	Distance (Å)	E (kcal/mol)	Dock. score (kcal/mol)	RMSD
*β-lactamases (PDB: 1CK3) protein E. coli*							
**10a**	N 3	OG SER235	H-donor	2.96	− 0.8	− 5. 1827	0.8981
	6-ring	CB ALA237	π-H	4.64	− 0.6		
	5-ring	NH1 ARG244	π -cation	4.14	− 3.4		
**11a**	N 7	ND2 ASN132	H-acceptor	3.15	− 2.5	− 5. 8781	0.9537
	6-ring	CB ALA237	π-H	4.37	− 0.9		
**11b**	N 7	ND2 ASN132	H-acceptor	3.16	− 2.0	− 5.8148	0.9530
	6-ring	ND2 ASN132	π-H	4.70	− 0.5		
	6-ring	CB PRO167	π-H	4.29	− 0.6		
	6-ring	CB ALA237	π-H	4.53	− 0.6		
*β-lactamases (PDB: 6MU9) protein B. megaterium*							
**10a**	N 3	O SER147	H-donor	3.05	− 4.9	− 5.1059	1.5201
	C 12	6-ring TYR122	H-π	3.92	− 0.5		
	6-ring	CA GLY255	π –H	4.03	− 0.6		
**11a**	5-ring	CA GLY255	π-H	4.00	− 0.5	− 6.1709	0.8763
**11b**	C 28	OE1 GLU188	H-donor	3.51	− 0.5	− 6.3742	1.3257
	6-ring	CB PRO184	π-H	4.14	− 0.6		
	6-ring	CB ALA254	π-H	4.56	− 0.5		
*β-lactamases (PDB: 6W2Z) protein B. Subtilis*							
**10a**	N 3	OD1 ASN 149	H-donor	2.93	− 1.6	− 5.2092	1.2156
	O 8	ND2 ASN 149	H-acceptor	3.00	− 0.5		
	6-ring	OG SER 147	π-H	4.22	− 0.8		
**11a**	N 7	OG SER252	H-acceptor	3.03	− 1.3	− 5.8862	0.7678
	5-ring	NH1 ARG260	π -cation	3.82	− 1.4		
**11b**	N 7	OG SER147	H-acceptor	3.41	− 7.9	− 5.8825	1.3683
	5-ring	OG SER252	π-H	4.41	− 1.0		

## Experimental section

### Materials and instrumentation

All MP were determined using the Akofler Block instrument. IR spectra (KBr) were obtained on an FTIR 5300 spectrometer (υ, cm⁻^1^). The ^1^H-NMR spectra were recorded on a Varian Gemini spectrometer at 400 MHz. MS were obtained with a 1000 EX mass spectrometer at 70 eV. The compounds 3-phenylisoxazol-5(4*H*)-one **2** and 4-acetyl-3-phenylisoxazol-5(4*H*)-one **3** were prepared following established literature methods as referenced [46, 47].

### Synthesis and spectroscopic characterization

#### Preparation of 4-methyl-6-oxo-3-phenyl-6*H*-pyrano[3,2-*d*]isoxazole-5-carbonitrile (4)

For 12h, a combination of **3** (0.01 mol) and ethyl cyanoacetate (0.01 mol) was refluxed in the presence of 30 mL of NaOEt. After allowing the mixture to cool, it was poured onto crushed ice and acidified with HCl to pH 2–3. After being filtered out, the separated solid was cleaned with H_2_O and it crystallized from EtOH to yield **4** as brown crystals; Yield 80%; MP = 281–283°C; IR (ν, cm^−1^): 3059 (CH-aromatic), 2981 (CH-aliphatic), 2206 (CN), 1720 (CO); ^1^H-NMR (DMSO-d_6_): *δ* 2.65 (s, 3H, CH_3_), 6.96–7.97 (m, 5H, aromatic H); MS: m/z (%) 252 (M^+^). Anal. for C_14_H_8_N_2_O_3_ (252). Calcd: C, 66.67; H, 3.20; N, 11.11%. Found: C, 66.73; H, 3.27; N, 11.18.

#### Preparation of 6-amino-8-hydroxy-5-oxo-1-phenyl-5*H*-isochromeno[4,3-*d*]isoxazole-7-carbonitrile (5)

A mixture of **4** (0.01 mol) and ethyl cyanoacetate (0.01 mol) in the presence of 30 mL of NaOEt was refluxed for 7h. After allowing the mixture to cool, it was poured onto crushed ice and acidified with HCl to pH 2–3. After being filtered out, the solid was washed with H_2_O, and crystallized from EtOH to yield **5** as brown crystals; Yield 77%; MP > 300°C (decomposition); IR (ν, cm^−1^) 3427–3400 (OH/NH_2_), 3060 (CH-aromatic), 2205 (CN), 1723 (CO); ^1^H-NMR (DMSO-d_6_): *δ* 7.10–7.53 (m, 8H, aromatic H and NH_2_), 8.31 (s, 1H, OH); MS: m/z (%) 319 (M^+^). Anal. for C_17_H_9_N_3_O_4_ (319). Calcd: C, 63.95; H, 2.84; N, 13.16%. Found: C, 63.99; H, 2.91; N, 13.22%.

#### Preparation of 4-(2-(dimethylamino)vinyl)-6-oxo-3-phenyl-6*H*-pyrano[3,2-*d*]isoxazole-5-carbonitrile (6)

The mixture of **4** (0.01 mol) and DMF-DMA (0.01 mol) in 25 mL of dioxane was refluxed for 6h. The reaction mixture was left to cool. Filtered off, the separated solid was then cleaned with toluene and allowed to crystallize from EtOH to produce **6** as brown crystals; Yield 46%; MP = 180–182°C; IR (ν, cm^−1^): 3055 (CH-aromatic), 2978–2931 (CH-aliphatic), 2194 (CN), 1720 (CO); ^1^H-NMR (DMSO-d_6_):* δ* 3.34 (s, 6H, N(CH_3_)_2_), 6.61 (d, 1H, = CH, *J* = 12MHz), 7.10 (d, 1H, = CH, J = 16MHz), 7.20–7.95 (m, 5H, aromatic H); MS: m/z (%) 308 (M^+^ + 1). Anal. for C_17_H_13_N_3_O_3_ (307). Calcd: C, 66.44; H, 4.26; N, 13.67%. Found: C, 66.49; H, 4.33; N, 13.72%.

#### Preparation of 6-amino-1-phenyl-5*H*-isoxazolo[4',5':5,6]pyrano[3,4-*c*]pyridin-5-one (7)

A solution of** 6** (0.01 mol) and NH₄OH (3 mL) in EtOH (30 mL) was heated under reflux for 24h. After the reaction mixture was cooled, it was poured into crushed ice and acidified with HCl to pH 2–3. After the separated solid was filtered out, toluene was used to wash it and crystallized from toluene to produce **7** as brown crystals; Yield 60%; MP = 292–294°C; IR (ν, cm^−1^): 3424–3400 (NH_2_), 3059 (CH-aromatic), 1660 (CO); ^1^H-NMR (DMSO-d_6_): *δ* 6.60 (s, 2H, NH_2_), 7.36–7.50 (m, 7H, aromatic H); MS: m/z (%) 279 (M^+^). Anal. For C_15_H_9_N_3_O_3_ (279). Calcd: C, 64.52; H, 3.25; N, 15.05%. Found: C, 64.59; H, 3.31; N, 15.10%.

#### Preparation of 6-amino-1-phenyl-5*H*-thieno[3',4':4,5]pyrano[3,2-*d*]isoxazol-5-one (8)

To a solution of **4** (0.01 mol) in EtOH (30 mL) containing elemental sulfur (0.01 mol) and ( 0.1 mL) of piperidine was added. The mixture was then refluxed for 24h. After this period, the reaction mixture was transferred to 30 mL of cold H_2_O and acidified with HCl to a pH of 3. The resulting precipitate was filtered off and crystallized from dioxane to yield **8** as brown crystals; Yield 70%; MP = 200–202°C; IR (ν, cm^−1^): 3425–3400 (NH_2_), 3058 (CH-aromatic), 1661 (CO); ^1^H-NMR (DMSO-d_6_): *δ* 6.17 (s, 2H, NH_2_), 7.24–7.39 (m, 6H, aromatic H and CH-thiophene); MS: m/z (%) 286 (M^+^ + 2).). Anal. for C_14_H_8_N_2_O_3_S (284). Calcd: C, 59.15; H, 2.84; N, 9.85%. Found: C, 59.21; H, 2.91; N, 9.92%.

#### General procedure for preparation of 10a,b, and 11a,b

A solution of **9a,b** (0.01 mol) and N_2_H_4_ or PhNHNH_2_ (0.01 mol) in EtOH (30 mL) was stirred at RT for 5 min. The mixture was then transferred into crushed ice. The formed adducts were filtrated and crystallized from EtOH to yield the corresponding **10a,b** and** 11a,b.**

##### 4-Diphenyl-5H-pyrazolo[4,3-*d*]isoxazole (10a)

Reddish brown crystals; Yield 83%; MP = 252–254 °C; IR (ν, cm^−1^): 3379 (NH), 3059 (CH-aromatic); ^1^H-NMR (DMSO-d_6_) *δ* 7.12–8.12 (m, 10H, aromatic H), 8.72 (s, 1H, NH); MS: m/z (%) 261 (M^+^). Anal. for C_16_H_11_N_3_O (261). Calcd: C, 73.55; H, 4.24; N, 16.08%. Found: C, 73.61; H, 4.30; N, 16.11%.

##### 4-(4-Methoxyphenyl)-3-phenyl-5H-pyrazolo[4,3-d]isoxazole (10b)

Brown crystals; Yield 86%; MP = 244–246 °C; IR (ν cm^−1^): 3427 (NH), 3050 (CH-aromatic), 2932 (CH-aliphatic) cm^−1^; ^1^H-NMR (DMSO-d_6_): *δ* 3.83 (s, 3H, OCH_3_), 7.04–7.82 (m, 9H, aromatic H), 8.63 (s, 1H, NH); MS: m/z (%) 291 (M^+^). Anal. for C_17_H_13_N_3_O_2_ (291). Calcd: C, 70.09; H, 4.50; N, 14.42%. Found: C, 70.13; H, 4.55; N, 14.46%.

##### 3,4,6-Triphenyl-6*H*-pyrazolo[4,3-*d*]isoxazole (11a)

Beige crystals; Yield 81%; MP = 160–162 °C; IR (ν cm^−1^): 3055 (CH-aromatic); ^1^H-NMR (DMSO-d_6_) *δ* 6.53–8.32 (m, 15H, aromatic H); MS: m/z (%) 337 (M^+^). Anal. for C_22_H_15_N_3_O (337). Calcd: C, 78.32; H, 4.48; N, 12.46%. Found: C, 78.38; H, 4.53; N, 12.52%.

##### 4-(4-Methoxyphenyl)-3,6-diphenyl-6*H*-pyrazolo[4,3-*d*]isoxazole (11b)

Gray crystals; Yield 80%; MP = 180–182 °C; IR (ν, cm^−1^): 3050 (CH-aromatic), 2924 (CH-aliphatic); ^1^H-NMR (DMSO-d_6_): *δ* 3.86 (s, 3H, OCH_3_), 6.90–7.72 (m, 14H, aromatic H); MS: m/z (%) 367 (M^+^). Anal. for C_23_H_17_N_3_O_2_ (367). Calcd: C, 75.19; H, 4.66; N, 11.44%. Found: C, 75.23; H, 4.69; N, 11.50%.

#### General procedure for preparation of 14a,b

NH_4_OAc (1 mmol), malononitrile (1 mmol), and **12a,b** (1 mmol) in an oil bath at 100 °C were mixed and fused for 10 min. After treating the resulting solid residue with EtOH, it was filtered out and crystallized from EtOH to produce the corresponding **14a,b**.

##### 6-Imino-3,5-diphenyl-5,6-dihydroisoxazolo[4,5-*c*]pyridazine-7-carbonitrile (14a)

Brown crystals; Yield 79%; MP = 272–274 °C; IR (ν, cm^−1^) 3311 (NH), 3061 (CH-aromatic), 2204 (CN); ^1^H-NMR (DMSO-d_6_): *δ* 7.31–7.95 (m, 10H, aromatic H), 9.90 (s, 1H, NH); MS: m/z (%) 313 (M^+^). Anal. for C_18_H_11_N_5_O (313). Calcd: C, 69.00; H, 3.54; N, 22.35%. Found: C, 69.08; H, 3.59; N, 22.40%.

##### 6-Imino-5-(4-methoxyphenyl)-3-phenyl-5,6-dihydroisoxazolo[4,5-*c*]pyridazine-7-carbonitrile (14b)

Brown crystals; Yield 77%; MP = 230–232 °C; IR (ν, cm^−1^): 3331 (NH), 3061 (CH-aromatic), 2929 (CH-aliphatic), 2194 (CN); ^1^H-NMR (DMSO-d_6_): *δ* 3.85 (s, 3H, OCH_3_), 7.38–8.03 (m, 9H, aromatic H), 8.46 (s, 1H, NH); MS: m/z (%) 343 (M^+^). Anal. for C_19_H_13_N_5_O_2_ (343). Calcd: C, 66.47; H, 3.82; N, 20.40%. Found: C, 66.51; H, 3.86; N, 20.45%.

### DFT studies and frontier molecular orbital analysis

The computations were executed employing the Gaussian 09W software, which integrates the DFT package utilizing the B3LYP exchange–correlation method. The 6-311G (d, p)* basis sets were applied for the derivatives [61, 62]. Visualization of the molecular structures was facilitated through the Gauss View 6 software [63–65].

### Antimicrobial testing

Antimicrobial activity was assessed using the disc diffusion method against *E. coli*, *B. megaterium*, *B. subtilis*, and *T. harzianum*. Cultures were grown, and microbial suspensions were applied to appropriate agar plates. Compounds were applied on sterile paper discs and placed on the agar. Plates were incubated, and inhibition zones were measured. Ampicillin and Clotrimazole served as controls. MIC values were determined via the broth microdilution method in a 96-well plate setup. The lowest concentration of compound that inhibited growth defined the MIC [66].

### Antioxidant activity via DPPH assay

The antioxidant activity of **11a,b** was screened using the DPPH assay [67]. Initially, 8 mg of DPPH reagent was dissolved in 100 mL of methanol to achieve a concentration of 80 µL/mL. In a 96-well microplate, 100 µL of DPPH reagent was mixed with 100 µL of various concentrations of **11a**,**b** (1000, 500, 250, 125, 62.5, 31.25, 15.62, and 7.81 µg/mL). The mixture was incubated at RT for 30 min. After incubation, the absorbance was measured at 490 nm using an ELISA reader (TECAN, Groding, Austria), with 100% MeOH as the control. The ability of each compound to scavenge DPPH radicals was calculated using the following formula:

$$ {\mathrm{DPPH}}\;{\mathrm{scavenging}}\;{\mathrm{activity}} = \frac{{{\text{control absorbance }}{-}{\text{different compounds absorbance}}}}{{\text{control absorbance}}}{ } \times 100 $$

The antioxidant activity of the ascorbic acid, **11a,b** was measured as DPPH radical scavenging activity %, and the IC_50_ DPPH values (the concentration of the sample required to inhibit 50% of DPPH radicals) were determined.

### Swiss ADME predictions

The physicochemical, pharmacokinetic, and drug-like of **4**^_^**8**, **10a,b**, **11a,b**, and **14a,b** were evaluated using the Swiss ADME computational tool [68, 69]. Optimized molecular structures, prepared using molecular modeling software, were inputted as SMILES notations to predict lipophilicity (iLOGP, XLOGP3), water solubility (ESOL Log S, ESO solubility), and pharmacokinetic properties, including GI absorption, BBB permeability, and cytochrome P450 enzyme inhibition. The tool also assessed skin permeation and compliance with various drug-likeness rules (Lipinski, Ghose, Veber, Egan, Muegge), providing a bioavailability score for each compound [70–72]. This comprehensive computational analysis aids in the preliminary screening of compounds, streamlining the drug development process by identifying promising candidates for further empirical testing.

### In silico molecular docking simulation

A molecular docking study was conducted using the DFT geometrically optimized structures of compounds **4**^_^**8**, **10a,b**, **11a,b**, and **14a,b** with the MOE 2015.10 program. The crystal structures of three different types of G- and G + bacteria, designated as PDB: 1CK3, 6MU9, and 6W2Z, served as the target protein receptors for β-lactamases and were obtained from the Protein Data Bank (http://www.rcsb.org/pdb/). During the docking process, hydrogen atoms were added, water molecules were removed, atomic charges were specified, and the MMFF94x force field was employed to minimize energy.

## Conclusions

The research successfully synthesized novel isoxazolone derivatives **4**^_^**8**, **10a,b**, **11a,b**, and **14a,b**, and their structural integrity was confirmed using various spectroscopic techniques. These analyses ensured that the compounds were pure and structurally sound. Antimicrobial tests revealed that all tested derivatives exhibited moderate to good activity against various bacterial and fungal pathogenic strains, indicating their broad-spectrum efficacy. Among these compounds, **11a**,**b** were particularly notable for their significant concentration-dependent antioxidant activity. Compound **11b** showed the highest efficacy at lower concentrations, although both compounds were less effective than ascorbic acid.

Molecular docking studies provided valuable insights into the mechanism of action of these derivatives. The studies highlighted strong interactions between the isoxazolone derivatives and key bacterial enzymes crucial for cell wall synthesis. These interactions suggest that the compounds could effectively inhibit bacterial and fungal growth and proliferation, offering a promising new avenue for combating microbial resistance. Furthermore, DFT studies elucidated the electronic properties of the isoxazolone derivatives, confirming their chemical stability and potential efficacy as antimicrobial agents. These findings underscore the promising nature of these compounds for further development and optimization in pharmaceutical applications.

## Data Availability

No datasets were generated or analyzed during the current study.
